# Engineering chromosome rearrangements in cancer

**DOI:** 10.1242/dmm.049078

**Published:** 2021-09-29

**Authors:** Salvador Alonso, Lukas E. Dow

**Affiliations:** 1 Sandra and Edward Meyer Cancer Center, Weill Cornell Medicine, New York, NY 10021, USA; 2 Department of Medicine, Memorial Sloan Kettering Cancer Center, New York, NY 10021, USA; 3 Department of Medicine, Weill Cornell Medicine, New York, NY 10065, USA

**Keywords:** CRISPR, Cancer, Chromosomal rearrangements, Fusion oncogenes

## Abstract

The identification of large chromosomal rearrangements in cancers has multiplied exponentially over the last decade. These complex and often rare genomic events have traditionally been challenging to study, in part owing to lack of tools that efficiently engineer disease-associated inversions, deletions and translocations in model systems. The emergence and refinement of genome editing technologies, such as CRISPR, have significantly expanded our ability to generate and interrogate chromosomal aberrations to better understand the networks that govern cancer growth. Here we review how existing technologies are employed to faithfully model cancer-associated chromosome rearrangements in the laboratory, with the ultimate goal of developing more accurate pre-clinical models of and therapeutic strategies for cancers driven by these genomic events.

## Abstract (precis)

**Summary:** Chromosome rearrangements can be potent cancer drivers and effective therapeutic targets. Here, we review how genome-editing technologies can be exploited to engineer and study complex structural variants, and identify new treatment options.

## Introduction

Despite significant investments in cancer research, cancer death rates over the past 20 years have only seen modest annual decreases of 1.8% for men and 1.4% for women (Henley et al., 2020). Cancer therapies represent ∼25% of all clinical trials in the US but <5% of all investigational drugs are ultimately approved for patient use by the FDA (Kola and Landis, 2004). The use of disease models that do not faithfully recapitulate human cancer has been partly blamed for the high rate of attrition regarding compounds that enter clinical trials and for the failure to translate scientific advances from bench to clinic (Sharpless and Depinho, 2006). Given the finite research resources, advancing technologies that more accurately mimic human disease and – at the same time – are simple, efficient and cheap, is a critical goal for driving more-effective pre-clinical studies. As our understanding of the molecular basis for cancer expands, laboratory-based models will play an even more crucial role in characterizing recurrent genetic mutations and validating targets for precision medicine approaches, ultimately narrowing the gap between preclinical and clinical scientific findings.

Historically, modeling chromosomal rearrangements and other structural variants in the laboratory has relied on transgenic approaches, in which gene products – often fusion proteins – are overexpressed under the control of an exogenous promoter (Shtivelman et al., 1985; Heisterkamp et al., 1990, 1991; Adams et al., 1985). Although these technologies are simple and efficient, and have enabled several important basic discoveries, they do not faithfully recapitulate the events that occur during tumorigenesis. Other traditional approaches that are based on homologous recombination accurately model endogenous rearrangements but their technical complexity and low efficiency makes them an impractical tool to characterize the hundreds of structural variants that are being identified with increasing speed (Box 1). The emergence of new genome-editing technologies, in particular CRISPR, has drastically increased our ability to mirror the complexity of human disease, offering new opportunities to advance our understanding of cancer biology and, ultimately, develop more-effective treatments.

**Box 1. Cataloging structural variants**

The exponential increase of next-generation sequencing (NGS) technologies has enabled the use of high-throughput genomic analysis in patient care and the diagnosis of actionable genomic changes. For instance, established clinical diagnostic tests, such as MSK-IMPACT are designed to detect known fusions of a small subset of genes, i.e. ALK, ROS1 and RET. Identification of other clinically actionable fusions is possible through both DNA- and RNA-based methods using direct amplification or capture-based sequencing (Benayed et al., 2019; Reeser et al., 2017; Heydt et al., 2021). For mutation discovery, research studies often employ transcriptome (RNAseq), whole-exome (WES) or whole-genome sequencing (WGS), which enable the identification of novel cancer-associated chromosome rearrangements (Seshagiri et al., 2012; Ju et al., 2012). RNAseq is particularly effective to identify expressed fusion products and can pinpoint uncharacterized drivers (Benayed et al., 2019). WGS, although more expensive, allows the identification of structural variants in non-coding regions (Rheinbay et al., 2020).

## Biology of chromosomal rearrangements

In 1960, David Hungerford and Peter Nowell first described that cancer cells from patients with chronic myeloid leukemia (CML) had an abnormally short chromosome 22 (Nowell and Hungerford, 1960). This represented the first cytogenetic defect linked to cancer and was named the Philadelphia chromosome after the city in which it was discovered (Nowell, 2007). Analysis of various tumor types in the years that followed revealed that most cancers are associated with chromosomal rearrangements that were more extensive as the disease progressed (Sandberg, 1966). However, it was unknown whether these aberrations are a cause or a consequence of the oncogenic process. As cytogenetic and molecular techniques improved over the next two decades, subsequent studies revealed that the Philadelphia chromosome results from translocation of chromosomes 9 and 22, generating a fusion tyrosine kinase protein between the breakpoint cluster region (BCR) and the tyrosine-protein kinase ABL1 (BCR-ABL). Ultimately, these pivotal studies led to the discovery of imatinib, the first targeted therapy approved for cancer treatment (Druker et al., 2001).

Chromosomal rearrangements are large genomic alterations that result from double-strand DNA breaks (DSBs) at two different loci, which are then aberrantly repaired by non-homologous end joining (Richardson and Jasin, 2000). Chromosomal rearrangements are arbitrarily defined as involving ≥50 base pairs; they are classified as balanced when there is an even exchange of genetic material between two loci, such as reciprocal translocations and inversions, and as unbalanced when parts of a chromosome are lost or gained, such as insertions, duplications and deletions (Table 1). These rearrangements drive tumor growth by disrupting tumor-suppressor genes, altering gene copy number, creating oncogenic fusion proteins or juxtaposing a gene with the regulatory elements of another gene (Li et al., 2020).

**Table 1.**
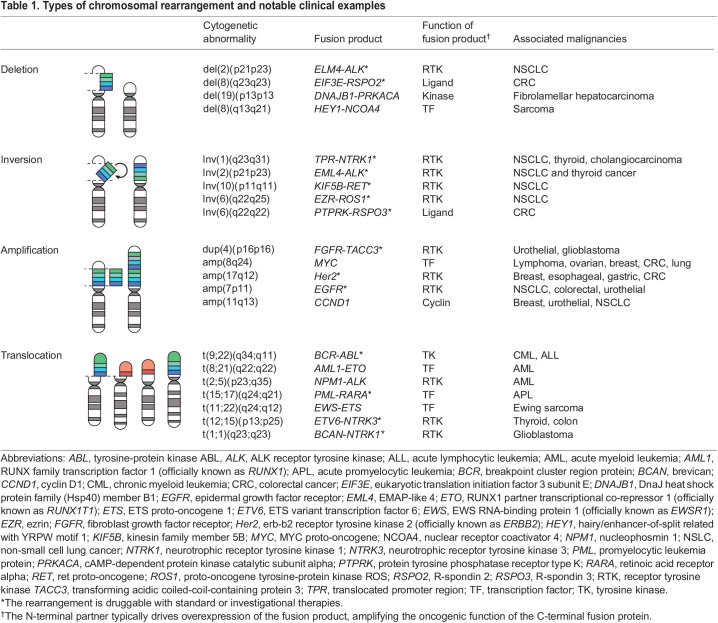
Types of chromosomal rearrangement and notable clinical examples

In recent years, the exponential increase in cancer genome sequencing has led to the identification of thousands of novel recurrent chromosomal rearrangements (see Mitelman Database of Chromosome Aberrations and Gene Fusions in Cancer; Box 1). These discoveries have led to groundbreaking treatments in select groups of patients. For instance, the use of small-molecule kinase inhibitors substantially improved treatment-response rates in patients with ALK receptor tyrosine kinase [*ALK*- (Kwak et al., 2010; Solomon et al., 2014)], *RET*- (Drilon et al., 2020) and *ROS1*-rearranged cancers (Shaw et al., 2014). Although such rearrangements – which drive the expression of constitutively active kinases, are of particular interest as they represent ‘druggable targets’, the oncogenic potential of the vast majority of recurrent structural variants remains untested.

For patients whose tumors harbor select oncogenic rearrangements, large randomized clinical trials have demonstrated improved outcomes after treatment with small-molecule inhibitors compared with chemotherapy. In the PROFILE 1014 trial, treatment with crizotinib was associated with longer progression-free survival (PFS) (10.9 months versus 7.0 months *P*<0.001) and improved response rates (74% versus 45%, *P*<0.001) among treatment-naïve patients diagnosed with *ALK*-rearranged non-small cell lung cancer (NSCLC) compared with patients who had received chemotherapy (Solomon et al., 2014). Similarly, the ASCEND-5 trial showed a significant improvement in PFS with ceritinib compared to chemotherapy in patients with *ALK*-rearranged NSCLC who had previously received crizotinib (5.4 months versus 1.6 months, *P*<0.001) in (Shaw et al., 2017). Randomized trials evaluating small-molecule inhibitors in patients diagnosed with ROS1-, NTRK1- and RET-rearranged cancer are ongoing but preliminary phase 1 and phase 2 studies have shown promising results, with improved outcomes compared to historical controls undergoing chemotherapy (Shaw et al., 2014; Drilon et al., 2018; 2020).

## Rarely found, rarely studied − the importance of engineering rearrangements

The study of cancer-associated mutations has often relied on patient-derived cell lines that carry a particular genotype. Although this strategy has facilitated the characterization of common oncogenic drivers associated with hematologic malignancies, for which patient samples are more easily accessible, efforts to study rare or diverse large-scale genomic events in carcinomas are often hampered by the lack of clinical specimens. Genetically engineered preclinical models provide a platform to study rare oncogenic drivers and offer some advantages over the traditional patient-derived systems. In particular, the ability to build models with any combination of cooperating events expands the ‘genetic space’ in which fusions can be investigated, and provides a platform to more thoroughly test new therapies. Furthermore, the generation of chromosome rearrangements in murine models enables studying cancer initiation and progression in the context of immunocompetent hosts.

Preclinical models may also provide proof-of-concept to test tailored therapeutic strategies in subgroups of patients for whom standard therapies do not exist or are ineffective. For instance, cancers harboring *ROS1* fusions often respond poorly to standard chemotherapy but are exquisitely sensitive to small-molecule kinase inhibitors (Solomon et al., 2014). Securing a meaningful number of clinical specimens to identify prognostic and predictive biomarkers would be challenging, as *ROS1* rearrangements are present in only 1-2% of all NSCLC cases. The use of preclinical models for these and other relatively rare but clinically significant alterations may accelerate the understanding of rare genomic events, and the development of novel therapeutic strategies (Arai et al., 2013). Although the individual incidence of cancers driven by specific gene fusions is low, collectively, they represent a high number of patients who may gain significant clinical benefit.

## Traditional tools to model chromosomal rearrangements

All chromosomal rearrangements – translocations, inversions, deletions and duplications – require the induction of DSBs at two separate loci and joining of otherwise unrelated genomic fragments. Not surprisingly, the efficient induction of specific chromosome rearrangements in the laboratory has proven difficult. Traditional approaches based on ectopic transgene expression, homologous recombination or Cre-loxP (Table 2) either poorly model endogenous rearrangements or are too inefficient to allow rapid characterization of newly identified cancer-associated mutations (Torres et al., 2014; Collins et al., 2000; Piganeau et al., 2013). Since 2014, older technologies have largely been replaced with CRISPR-based strategies that offer efficiency, simplicity and flexibility. In the following sections, we discuss the advantages and limitations of different genome-editing technologies for engineering chromosome rearrangements.

**Table 2.**
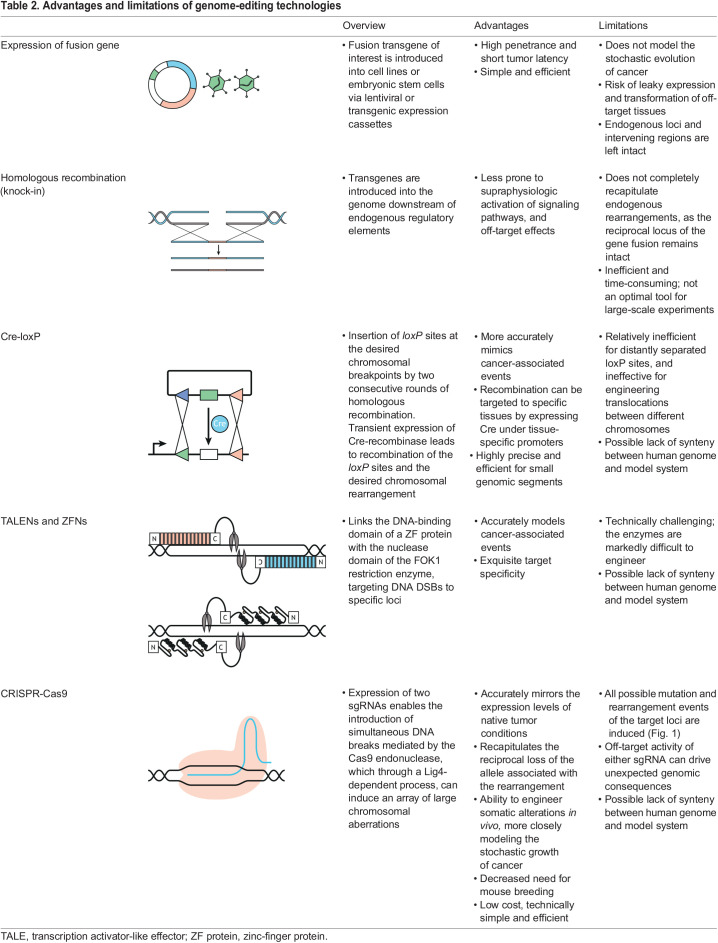
Advantages and limitations of genome-editing technologies

### Fusion gene expression

A large proportion of chromosome rearrangements result in the *de novo* generation of gene and protein fusions. As such, many efforts to model gene fusions have involved the expression of fusion protein-encoding cDNAs from heterologous promoters. In these systems, the fusion transgene of interest is introduced into cell lines or embryonic stem cells via lentiviral or transgenic expression cassettes. The approach was first used in the 1980s and 90s to engineer leukemia and lymphoma models driven by the *IGH-MYC* and *BCR-ABL* translocations, and quickly became the most widely used technique to model chromosomal rearrangements (Shtivelman et al., 1985; Heisterkamp et al., 1990, 1991; Adams et al., 1985). A major advantage of this system is its simplicity: virtually any oncogene or gene fusion can be cloned into an expression vector and rapidly employed to engineer transgenic cell lines or mice. For *in vivo* experiments, the ectopic transgenic method offers the added benefits of high penetrance and short tumor latency, cutting research costs by allowing the experimental cohorts to be maintained at a reasonable size (Sharpless and Depinho, 2006). For example, Soda and colleagues engineered mice that ectopically express the ELM4-ALK fusion protein in alveolar epithelial cells under the control of the surfactant C promoter (Soda et al., 2008). A few weeks after birth, ELM4-ALK transgenic mice developed hundreds of pulmonary nodules, allowing researchers to rapidly and reproducibly test the effect of small-molecule kinase inhibitors in brief and simple experiments (Soda et al., 2008).

Highly penetrant and aggressive phenotypes, by contrast, come with the trade-off of poorly recapitulating the stochastic evolution of human cancer, as the emergence of synchronous and multifocal large tumors is likely to blunt microenvironmental interactions and the effect of secondary oncogenic events (Sharpless and Depinho, 2006). These features are a consequence of what is, perhaps, the main limitation of transgenic approaches – supraphysiologic oncogene expression that does not mimic the tumor context. For instance, the pioneering mouse models of *BCR*-*ABL* leukemia were associated with embryonic or early postnatal lethality, a stark contrast to the indolent behavior of CML in humans (Heisterkamp et al., 1990, 1991; Sawyers, 1999). Similarly, mouse models of *MYC*-rearranged B-cell lymphoma and ALK-rearranged NSCLC displayed highly penetrant phenotypes, precluding the study of the stochastic events driving cancer growth (Adams et al., 1985; Soda et al., 2008). Selecting a tissue-specific promoter can help limit the effects to the cellular compartment of interest, but even transient or low-level transgene expression carries the risk of transforming off-target tissues (Chiarle et al., 2003).

One approach to limit uniform tissue transformation and unwanted transgene expression in non-target tissues is a ‘mosaic’ or somatic introduction of gene fusions. In these models, somatic cells may be transduced *ex vivo* and then transplanted into syngeneic recipients (Zuber et al., 2009; Lange et al., 2003), thus allowing temporal control of disease onset and facilitating the development of more-complex genotypes by introducing multiple oncogenes or inactivating mutations at once. Although this approach does overcome several limitations associated with germline transgenic methods, supraphysiologic expression of the transgene remains the main concern, with the potential to transform unintended cellular compartments. For example, two independent research groups detected B-cell malignancies in mice upon transplantation of HSCs that express the NPM-ALK fusion protein, which – in humans – is exclusively observed in T-cell anaplastic large-cell lymphoma (Lange et al., 2003; Kuefer et al., 1997). Moreover, the transgenic somatic approach only had success in the hematopoietic system that allows for *ex vivo* transduction of stem cells.

A further limitation of the ectopic transgenic approach is that the endogenous genes remain unmodified and the transgenes, therefore, do not entirely recapitulate the oncogenic insult. For example, fusions driven by chromosomal deletions can involve the heterozygous loss of a large number of genes, whereas inversions and translocations can disrupt ‘partner’ genes or regulatory elements, such as enhancers, within intervening regions. Classic examples of this phenomenon are nucleophosmin 1 (*NPM1*) and *PTPRK*, putative tumor suppressor genes that are recurrently fused to *ALK1* and *RSPO3*, respectively (Grisendi et al., 2006; Chang et al., 2020; Shimozato et al., 2015). Disruption of *PTPRK* was shown to accelerate tumor growth through the phosphorylation of the cancer stem cell marker PROM1 and through activation of AKT signaling (Shimozato et al., 2015). Therefore, overexpression of *RSPO3* and loss of *PTPRK* could confer a double hit in colorectal cancer (CRC) harboring the *RSPO3-PTPRK* fusion. Similarly, loss of NPM1 may destabilize tumor suppressor proteins, such as p53 and synergize with ALK overexpression to promote tumor growth in lymphomas with *NPM1-ALK* fusions (Grisendi et al., 2006).

One final, albeit rare issue with transgenic approaches is that they do not recapitulate the reciprocal product (e.g. ABL-BCR) of the translocation. Although expression of a reciprocal fusion product only occurs in some situations (Grisendi et al., 2006), there are cases in which both gene fusions are expressed and contribute to cancer growth. For example, both products of the reciprocal translocation *t*(11;17), *PLZF-RARα* and *RARα-PLZF*, are necessary to induce acute promyelocytic leukemia in mice (He et al., 2000).

### Knock-in and conditional approaches

In contrast to the expression of fusion cDNAs under the control of heterologous promoters, transgenes may be introduced into the genome downstream of endogenous regulatory elements, thus more closely reflecting the expression pattern of cancer-linked gene fusions. One of the first examples was the generation of the Eµ-Myc mouse, created by transgenic insertion of the *MYC* protooncogene downstream of the immunoglobulin heavy chain enhancer (Adams et al., 1985), mimicking the translocation of *MYC* observed in almost all Burkitt lymphomas (Taub et al., 1982). In this model, the fusion cassette is not positioned at the endogenous Ig locus, MYC expression is still restricted to B cells and these mice form B-cell lymphomas. Corral and colleagues employed a similar strategy to characterize the role of *Mll-AF9* in acute leukemia (Corral et al., 1996). Despite the presence of the gene fusion in the germline, mice exclusively developed acute myeloid leukemia (AML), recapitulating the spectrum of human cancers with the translocation *t*(9;11). Subsequent studies employed homologous recombination of transgenes to characterize the role of BCR-ABL in CML and acute lymphoblastic leukemia (Castellanos et al., 1997; Foley et al., 2013).

The main advantage of this knock-in approach is that gene expression is controlled through endogenous regulatory elements and, therefore, is less prone to supraphysiologic pathway activation and off-target tissue effects. However, this approach does not completely recapitulate rearrangements, as the reciprocal locus of the gene fusion remains intact. The technique is also time-consuming and not ideal for large-scale experiments or rapid interrogation of novel rearrangements.

The Cre-loxP system more accurately mimics cancer-associated events by creating rearrangements between two separate target loci (Van Deursen et al., 1995). In this approach, *loxP* sites are inserted at the desired chromosomal breakpoints by two consecutive rounds of homologous recombination. Transient expression of Cre-recombinase leads to recombination of the *loxP* sites and the desired chromosomal rearrangement. By expressing Cre-recombinase under the control of a tissue-specific promoter, recombination events can be targeted to specific tissues. Conditional expression of Cre-recombinase, e.g. by using tetracycline- or tamoxifen-regulated alleles, can be used to time events of recombination. Cre-loxP recombination is remarkably precise and efficient for the deletion of small genomic segments, e.g. floxed KO alleles, but is relatively inefficient for distantly separated *loxP* sites and generally ineffective when modeling translocations between different chromosomes (Yu and Bradley, 2001). Despite this issue, the Cre-loxP system has been employed to model recurrent gene fusions observed in sarcomas and hematologic malignancies (Keller et al., 2004; Collins et al., 2000; Smith et al., 1995; Buchholz et al., 2000; Forster et al., 2003; Drynan et al., 2005). However, with the exception of the *Mll-Enl* fusion leukemia mouse model (Forster et al., 2003; Drynan et al., 2005), the chromosomal rearrangements were not sufficient to induce malignancies in the engineered mice. The lack of malignant transformation is most probably related to low recombination efficiencies in target tissues and decreased expression of oncogenes compared with that in transgenic mouse models (Yu and Bradley, 2001).

Recently, Lowe and colleagues used the Cre-loxP system to engineer mouse models of AML and lymphoma with 17p deletion. Somatic heterozygous deletion of the mouse chromosome 11B3, a region syntenic to human 17p13 and encompassing the *Trp53* locus, resulted in a more-aggressive phenotype compared with homozygous loss of *Trp53* only (Liu et al., 2016). The aggressive phenotypes were the result of simultaneously deleted tumor suppressor genes on mouse chromosome 11B3, which underscores the selective advantage segmental deletions or other chromosomal rearrangements may confer to cancer cells due to the disruption of multiple genes. These findings further highlight the importance of accurately modeling these understudied oncogenic events.

## Chromosomal rearrangements through genome editing

### ZFNs and TALENs

Zinc-finger nucleases (ZFNs) link the DNA-binding domain of a customizable zinc finger protein with the nuclease domain of the FOK1 restriction enzyme (Urnov et al., 2010), targeting DNA DSBs to specific genomic loci. Like ZFNs, transcription activator-like effector (TALE) nucleases (TALENs) contain the nuclease domain of FOK1 but use TALE prokaryotic transcription factors as the DNA-binding domain. By simultaneously targeting two separate loci, TALENs and ZFNs have been employed to engineer the *Ewsr1*-*Fli1* and *Npm1*-*Alk* fusions implicated in Ewing sarcoma and anaplastic large-cell lymphoma, respectively (Piganeau et al., 2013). Because both ZFNs and TALENs require binding of two FOK1-linked proteins for each target locus, they have exquisite target specificity. However, these enzymes are also much more difficult to engineer and have quickly been overshadowed by the emergence of CRISPR-Cas9 tools.

### CRISPR-Cas9

Clustered regularly interspaced short palindromic repeats (CRISPR) is a programmable, RNA-guided genome-editing system that has completely revolutionized the field of cancer genetics owing to its low cost, ease of use and high efficiency. First identified as a crucial component of bacterial immunity against phage infection, the system was subsequently engineered to target alternate DNA sequences in bacteria (Jinek et al., 2012) and mammalian cells (Cong et al., 2013; Jinek et al., 2013; Mali et al., 2013; Ran et al., 2013). The functional unit is made up of a dual RNA complex or single guide RNA (sgRNA) and a CRISPR-associated endonuclease, usually CRISPR-associated protein 9 (Cas9). These two components form a ribonucleoprotein complex that scans the genome for complementary DNA sequences adjacent to small consensus sequences called protospacer adjacent motifs (PAMs) (Sternberg et al., 2014). Given sufficient DNA-RNA homology, the endonuclease domains of Cas9 mediate a DNA DSB (Cong et al., 2013; Jinek et al., 2013; Mali et al., 2013). Most importantly, targeting specificity is achieved by simply modifying a 17-20 bp sequence within the sgRNA, thus providing a means to engineer specific tools for multiple loci with relative ease.

Whereas Cas9-mediated DNA cleavage is often repaired by error-prone non-homologous end joining leading to small indels at the break site, expression of two sgRNAs enables the introduction of simultaneous DNA breaks that, through a Lig4-dependent process, can induce an array of large chromosomal aberrations (Li et al., 2015). In most cases, generating precise fusions of the two breakpoints is not crucial because the sgRNAs are usually engineered to target intronic regions. As a result, splicing of the exons will most often create the desired fusion transcript. Using this approach, multiple complex chromosomal rearrangements involved in hematological malignancies as well as lung, liver, brain and intestinal cancer have been engineered (Blasco et al., 2014; Maddalo et al., 2014; Li et al., 2015; Xue et al., 2014; Cook et al., 2017; Han et al., 2017).

CRISPR-Cas9 was first used to model recurrent oncogenic chromosomal rearrangements in cancer cell lines and primary cells, including the translocations *t*(11;22) and *t*(8;21) observed in Ewing sarcoma and AML, and the inversions inv(2)(p21p23) and inv(10)(p11q11) observed in NSCLC (Torres et al., 2014; Choi and Meyerson, 2014). Shortly after, Maddalo et al. and Blasco et al. used an *in vivo* somatic approach to engineer mouse models carrying *Eml4-Alk* (inversion) fusion-driven lung cancers via intratracheal instillation of recombinant adenoviruses (Maddalo et al., 2014; Blasco et al., 2014). Expression of Cas9 and sgRNAs in the endobronchial epithelium induced the endogenous inversion of chromosome 17, rearrangement of the *Elm4-Alk* loci and tumor growth with 100% penetrance (Maddalo et al., 2014; Blasco et al., 2014). The efficiency of the system in generating the *Elm4-Alk* fusion *in vivo* was estimated to be 1.5 rearrangements per 10^6^ cells (Blasco et al., 2014). As expected, *Alk-re*arranged tumors were sensitive to the small-molecule kinase inhibitor crizotinib (Maddalo et al., 2014). These pioneering studies demonstrated that CRISPR can be readily adapted to model cancer-associated chromosomal rearrangements, opening opportunities to better understand cancer initiation and progression, explore novel therapeutic strategies and investigate drivers of drug resistance *in vivo*. Subsequently, other groups adapted an optimized version of the same approach to engineer rearrangements in the liver and brain through tail-vein or intracranial injection of Cas9 and sgRNAs, respectively (Li et al., 2015; Xue et al., 2014; Cook et al., 2017).

For tissues that are not easily transduced *in vivo*, such as the intestine, inducible transgenic platforms allow temporal regulation of Cas9 expression to induce the desired rearrangements. For instance, Han and colleagues generated transgenic mice carrying a doxycycline (dox)-regulated Cas9 transgene and two sgRNAs targeting introns within *Eif3e* and *Rspo2* or *Ptprk* and *Rspo3* (Han et al., 2017). Treatment with dox induced the expected *Eif3e-Rspo2* deletion and *Rspo3-Ptprk* inversion. After 6 weeks, both models developed hyperproliferative and dysplastic lesions throughout the small intestine, although the phenotype was much less pronounced in *Eif3e-Rspo2* mice. It is worth noticing that this specific fusion is far less common in human CRC (Seshagiri et al., 2012; Sackstein et al., 2021) and is often associated with amplification of the 8q locus, suggesting a requirement for even further elevated expression of Rspo2 to induce tumor growth. Recently, Kawasaki et al. described the development of both *PTPRK-RSPO3* and *EIF3E-RSPO2* fusions in human colon organoids (Kawasaki et al., 2020). Unlike the murine model where only *Rspo3* fusions enabled organoid growth in RSPO-free medium (Han et al., 2017) both fusions enabled niche independence in the human organoids (Kawasaki et al., 2020). The precise functional difference between the two models is unclear but could reflect differences in the 5′ untranslated region of mouse and human *Rspo2* fusions that impact gene expression (Han et al., 2017). The polyps of the *Ptprk-Rspo3 in vivo* model were widespread in the small intestine and harbored at least one copy of the inversion in most of the tumor cells, suggesting a cell-intrinsic advantage of carrying the fusion. This observation contradicts an independent study in which a Cre-dependent *Rspo3* cDNA transgene was induced into LGR5^+^ intestinal stem cells and the resulting epithelial hyperproliferation was reportedly driven by paracrine secretion of the Rspo3 ligand (Hilkens et al., 2017). These conflicting findings underscore how subtle differences in genetic models can have different effects on disease phenotypes.

The simplicity of using CRISPR to create fusions has provided an incentive to characterize the oncogenic potential of newly identified and often very rare cancer-associated structural variants (Box 1). These new model systems can then be used to prospectively test sensitivity to existing or novel targeted therapies. In one example, Cook and colleagues sought to characterize multiple novel recurrent chromosomal rearrangements observed in high-grade gliomas (Cook et al., 2017). In one of the engineered models, intracranial injection of adenoviruses expressing Cas9 and paired sgRNAs led to the expected deletion on chromosome 3, and growth of high-grade gliomas carrying the *Bcan-Ntrk1* fusion. These tumors were sensitive to entrectinib, a pan-TRK inhibitor that has now been approved by the FDA for patients who have tumors harboring neurotrophic receptor tyrosine kinase (NTRK) gene fusions. By engineering an interstitial deletion in chromosome 3, the study provided proof of concept for using CRISPR somatic editing to model chromosomal rearrangements that do not result in gene fusions (Cook et al., 2017).

Using the same approach, Kastenhuber et al. (2017) applied CRISPR editing to develop a mouse model of fibrolamellar hepatocellular carcinoma. This extremely rare type of liver cancer primarily affects adolescents and young adults, and is invariably associated with a segmental deletion on chromosome 19, generating an in-frame fusion of *DNAJB1* and *PRKACA* (Honeyman et al., 2014). The authors used different genome-editing approaches to develop a series of mouse models and showed that tumor growth depends on the kinase domain of the fusion protein, paving the way for clinical trials employing small-molecule inhibitors (Abou-Alfa et al., 2021).

A CRISPR-based approach to model a chromosomal rearrangement overcomes several of the limitations associated with the more traditional strategies of transgene expression or homologous recombination. By targeting the endogenous loci of the rearrangement, the model accurately mirrors the expression levels observed in the native tumor conditions. In addition, it recapitulates the loss of the reciprocal allele associated with the rearrangement, which may also promote tumor growth. The ability to engineer somatic alterations *in vivo* – impossible with traditional approaches due to their poor recombination efficiencies – offers additional advantages. By targeting only a subset of cells, somatic engineering more closely mirrors the natural evolution and stochastic growth of human cancer. For example, somatic CRISPR models of *Alk*-rearranged lung cancer displayed more-indolent growth compared with those of transgenic germline systems (Blasco et al., 2014; Soda et al., 2008). Furthermore, a single Cas9/sgRNA vector can be readily adapted to model rearrangements or other cooperating mutations in different genetic backgrounds, without the need for mouse breeding, thereby, significantly decreasing research costs.

### Limitations of CRISPR-based genome editing

**Fig. 1.**
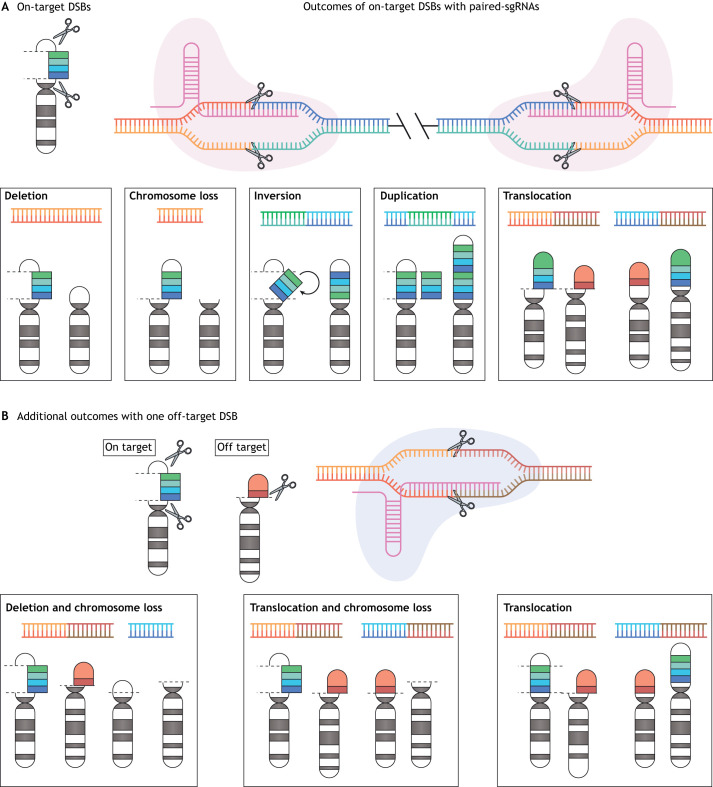
**Unintended rearrangements involving on-target and off-target loci upon CRISPR-based editing.** (A) All possible rearrangement events of the target loci – deletions, inversions and duplications – are induced following CRISPR-mediated double-strand DNA breaks (DSBs) with paired sgRNAs. Positive selection within a bulk population of gene-edited cells often drives the enrichment of oncogenic rearrangements. (B) When DSBs are induced in one or more off-target loci, the number of possible unintended rearrangements grows exponentially, including novel fusion events, loss of entire chromosome segments, dicentric and acentric chromosomes (not shown).

Widespread use of CRISPR over the past 8 years has led to significant improvements in its potency and specificity. However, some challenges remain to be addressed (Table 2). One caveat of the dual sgRNA targeting system is that, within a population of cells, all possible mutation and rearrangement events of the target loci are induced (Fig. 1). These include focal indels at each site, representing the majority of events, as well as inversions, deletions and duplications. Fortunately, in many cancer models, positive selection can drive the enrichment of an oncogenic lesion. For example, Cook and colleagues showed that expression of Cas9 and paired sgRNAs in neural stem cells led to both inversions and deletions on chromosome 3; yet, only clones harboring a deletion expressed the BCAN-NTRK1 fusion protein and drove tumor growth (Cook et al., 2017). However, for events with weak tumor-promoting potential, identifying relatively rare clones or interpreting data obtained from mixed populations can be challenging.

In addition to unintended rearrangements between the on-target loci, off-target activity of either sgRNA can drive unexpected and, possibly, deleterious consequences, including the generation of completely novel fusion events, dicentric chromosomes and loss of entire chromosome segments (Perez et al., 2017). If an sgRNA has multiple off-target loci, the number of possible unanticipated events grows exponentially, especially when dealing with aneuploid or hyper-diploid cancer cells (Fig. 1). Although the development of effective high-fidelity Cas9 nuclease variants can limit off-target activity (Kleinstiver et al., 2016; Zafra et al., 2018; Vakulskas et al., 2018), in many cases – even sgRNAs predicted to have high specificity – can target multiple genomic loci with near-identical sequences (Perez et al., 2017; Fu et al., 2013). The optimization of NGS-based tools may facilitate identification of off-target effects, and increase the specificity and reproducibility of CRISPR-based models (Wienert et al., 2020; Zuo et al., 2020). The final major limitation for the generation of chromosome rearrangement is not unique to CRISPR but a problem for all non-human models. Even with highly specific and active sgRNAs, some rearrangements seen in human disease are simply impossible to recreate in a model due to lack of synteny or lack of conservation of intron-exon boundaries across species.

## Future directions

Chromosome rearrangements are a frequent and diverse group of cancer-associated genetic events. Most importantly, rearrangements frequently drive the production of gene fusions that act as oncogenic drivers. Although the overall incidence of cancers harboring individual rearrangements is low, the aggregate represents a high number of patients. Thus, developing fast, flexible and cost-effective methods to characterize the increasing number of recurrent chromosomal rearrangements that are being identified each year is a crucial step toward realizing the goals of precision medicine (Li et al., 2020). As the examples above demonstrate, CRISPR technologies have been instrumental in engineering and characterizing chromosomal rearrangements that directly contribute to cancer growth through expression of fusion proteins or disruption of tumor suppressor genes. Yet, CRISPR tools also offer flexibility and efficiency to functionally characterize putative oncogenic structural variants that involve non-coding regions (Rheinbay et al., 2020; Fujimoto et al., 2016; Quigley et al., 2018). Such rearrangement within non-coding regions may mediate the upregulation of nearby oncogenes and downregulation of tumor suppressor genes by, for example, altering regulatory elements or non-coding RNAs (Rheinbay et al., 2020; Quigley et al., 2018; Fujimoto et al., 2016). Larger datasets and advances in sequencing technologies (Box 1) will continue to reveal new coding and non-coding structural variants, and CRISPR will play a central role in modeling and characterizing the impact of these changes – both in cancer and other genetic disorders that are linked to chromosome rearrangements.
